# Iodo Silanes as Superior Substrates for the Solid Phase Synthesis of Molecularly Imprinted Polymer Nanoparticles

**DOI:** 10.3390/polym14081595

**Published:** 2022-04-14

**Authors:** Stanislav S. Piletsky, Alvaro Garcia Cruz, Elena Piletska, Sergey A. Piletsky, Eric O. Aboagye, Alan C. Spivey

**Affiliations:** 1 Department of Chemistry, Molecular Sciences Research Hub, White City Campus, Imperial College London, London W12 0BZ, UK; a.c.spivey@imperial.ac.uk; 2 School of Chemistry, College of Science and Engineering, University of Leicester, Leicester LE1 7RH, UK; agc14@leicester.ac.uk (A.G.C.); ep219@le.ac.uk (E.P.); sp523@le.ac.uk (S.A.P.); 3 Department of Surgery and Cancer, Hammersmith Campus, Imperial College, London W12 0NN, UK; eric.aboagye@imperial.ac.uk

**Keywords:** molecularly imprinted polymers, solid phase synthesis, epidermal growth factor receptor

## Abstract

Current state-of-the-art techniques for the solid phase synthesis of molecularly imprinted polymer (MIP) nanoparticles typically rely on amino silanes for the immobilisation of template molecules prior to polymerisation. An investigation into commonly used amino silanes identified a number of problematic side reactions which negatively affect the purity and affinity of these polymers. Iodo silanes are presented as a superior alternative in a case study describing the synthesis of MIPs against epitopes of a common cancer biomarker, epidermal growth factor receptor (EGFR). The proposed iodo silane outperformed the amino silane by all metrics tested, showing high purity and specificity, and nanomolar affinity for the target peptide.

## 1. Introduction

The last thirty years have seen molecularly imprinted polymers (MIPs) progress from serving as simple affinity matrices in solid phase extraction to finding applications in sensors, assays, drug delivery and proteomics [1,2,3,4,5,6,7,8,9]. Despite this, the majority of MIPs described within more recent work suffer from the same issues as their historical predecessors: difficulty in removal of the template and a high level of non-specific binding due to the ‘polyclonal’ nature of the binding sites. In order to address these shortcomings, a new technique for MIP synthesis emerged, involving the immobilisation of template molecules onto a solid phase prior to performing polymerisation [10]. This allows for easy removal of template molecules, screening for high affinity polymers, and greater uniformity of binding sites [10]. Since the introduction of solid phase imprinting in 2013, the use of this technique has spread considerably, with Google Scholar now featuring over 600 papers referring to the use of solid phase synthesis of MIPs.

To date, the choice of solid phase substrate and the nature of the linker used for template immobilisation has not been thoroughly explored. Thus far, the assumption seems to have been that the solid phase acts as an ‘innocent bystander’ during MIP synthesis, serving to immobilize the template without taking part in reactions with monomers or initiators. The most common solid phases used for MIP synthesis are glass beads modified with various functional groups suitable for conjugation to template molecules, such as amines for amide formation and alkynes for click-coupling to azides [10,11,12]. The surface modifiers are invariably silanes, such as (3-aminopropyl)triethoxysilane (APTES), *N*-(6-aminohexyl)aminomethyltriethoxysilane (AHAMTES), or 2-propynyl [3-(triethoxysilyl)propyl]carbamate. In particular, amino silanes are commonly used due to their low price, commercial availability, and versatility for the immobilisation of templates possessing carboxylic acids, amines and thiols [4,13,14].

**Scheme 1:**
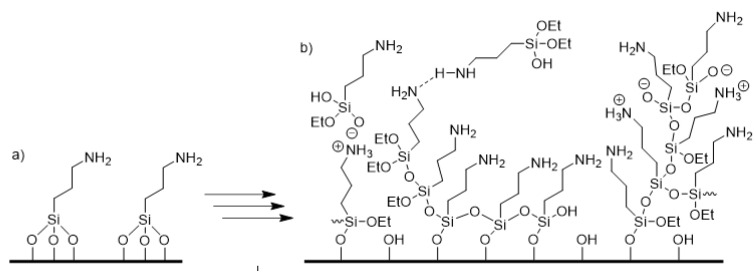
(**a**) APTES monolayer on surface of glass; (**b**) weakly bound APTES multilayers on the surface of glass. Adapted from Ref. [15].

These silanes suffer from a number of shortcomings. Notably, amino silanes are known to form weakly bound multilayers on the surface of glass (Scheme 1, Figure S1) [15]. The formation of multilayers is problematic because many of these groups will be lost during extensive washing prior to or following polymerisation, resulting in wasted template molecules and MIP contamination with both template and silane.

Furthermore, we have observed that amino silanes induce the decomposition of persulphates. Reactions between ammonium persulphate and two amino silanes (APTES and AHAMTES) were shown to initiate polymerisation of acrylamide even in the absence of common tertiary amine co-initiators, such as *N*′-tetramethyl ethylenediamine (TEMED) (Figure S2, Table S1). As ammonium persulphate is a common radical initiator for MIP polymerisation, the presence of amino silanes may, therefore, result in unintended side reactions and poorly controlled polymerisation.

This work demonstrates these limitations and proposes iodo silanes as a superior alternative to amino silanes via a case study involving the synthesis of MIP nanoparticles against epitopes of epidermal growth factor receptor (EGFR), a cancer biomarker of great clinical significance. The conjugation of EGFR peptides to iodo silane is straightforward, involving coupling to the thiol of a terminal cysteine under basic conditions. Though reactions between alkyl halides and thiols are commonplace, the use of iodo silanes for solid phase immobilisation of biological molecules is rare, with few examples in literature [16]. In contrast, amine silane coupling requires more steps including the use of expensive linkers, such as succinimidyl iodoacetate (SIA) [13]. The synthesised MIP nanoparticles showed excellent uniformity, affinity and specificity for the imprinted EGFR epitope (K_d_ = 2.3 nM).

## 2. Materials and Methods

### 2.1. Materials

All reagents used in this project were obtained from Sigma-Aldrich Company Ltd. (Poole, UK) or Thermo Fisher Scientific Ltd. (Loughborough, UK) unless otherwise stated. Acryloxyethyl thiocarbamoyl rhodamine B was purchased from Polysciences Europe GmbH (Hirschberg an der Bergstrasse, Germany). Peptides were synthesised by ZheJiang Ontores Biotechnologies Co., Ltd. (Hangzhou, China).

### 2.2. Preparation of APTES, AHAMTES and IPTMS Solid Phase

Glass beads (60 g) were boiled in NaOH (1 M, 100 mL) for 15 min, washed with water (5 × 200 mL), phosphate buffered saline (PBS, 10 mM phosphate buffer, 2.7 mM KCl, 137 mM NaCl, pH 7.4, 100 mL), water (5 × 200 mL) and acetone (2 × 100 mL). They were then dried by heating to 120 °C for 30 min. A solution of 2% (*v*/*v*) (3-aminopropyl)triethoxysilane (APTES) or N-(6-aminohexyl)aminomethyltriethoxysilane (AHAMTES) or (3-iodopropyl)trimethoxysilane (IPTMS) in dry toluene (60 mL) was added to the activated beads. Following overnight (16 h) incubation at 70 °C, the beads were washed with acetone (4 × 100 mL) and dried at 120 °C for 30 min.

### 2.3. Immobilisation of Peptide on AHAMTES Glass

AHAMTES-functionalised beads (60 g) were soaked in a large volume of water for 24 h in order to remove multilayers. The beads were then washed with acetone (4 × 100 mL) and dried at 120 °C for 30 min, then incubated with succinimidyl iodoacetate (SIA) (5 mg, 18 µmol) in anhydrous acetonitrile (25 ml) and incubated for 2 h under exclusion of light, before washing with acetonitrile (5 × 50 mL).

Ethylenediaminetetraacetic acid (EDTA) (74 mg, 500 µmol) was dissolved in phosphate buffered saline (PBS, 10 mM, 50 mL) and adjusted to pH 8.2 with sodium hydroxide. SIA-functionalised glass beads (60 g) and EGFR peptide (5 mg) were then added, and the mixture was incubated overnight protected from light. Mercaptoethanol (20 µL, 0.3 mmol) was then added and incubated for 2 h. The beads were then washed with water (3 × 200 mL) and acetone (1 × 100 mL) and allowed to dry.

### 2.4. Immobilisation of Peptide on IPTMS Glass

EGFR peptide (5 mg) was dissolved in borate buffer (pH 9.2, 30 mM sodium tetraborate, 25 mL), added to IPTMS-functionalised glass beads (60 g) and incubated overnight. Mercaptoethanol (20 µL, 0.3 mmol) was then added and incubated for 2 h. The beads were then washed with water (3 × 200 mL) and acetone (1 × 100 mL) and allowed to dry.

### 2.5. Peptide Density Measurement

Peptide density in the solid phase was measured using the Pierce Rapid Gold BCA Protein Assay Kit (Thermo Scientific, Waltham, MA, USA). As described in the Thermo Fisher Protein Assay Technical Handbook, Buffer A and Buffer B were mixed in a 50:1 ratio; 400 µL of this solution was mixed with 100 mg of peptide-coated glass beads, and the mixture was incubated at 37 °C with shaking for 30 min. The samples were allowed to cool, and 100 µL of the solution was transferred to each of the three wells in a 96 well microtiter plate. The absorbance of these wells was measured at 562 nm using a Hidex Sense plate reader (LabLogic, Sheffield, UK). A calibration curve was prepared by repeating this measurement using known concentrations of peptides.

### 2.6. MIP Synthesis

The following monomers were dissolved in water (50 mL): *N*-isopropyl acrylamide (20 mg, 180 µmol), *N*-tert-butylacrylamide (16.5 mg, 130 µmol), *N,N*’-methylene bis(acrylamide) (3 mg, 20 µmol), *N*-(3-aminopropyl)methacrylamide hydrochloride (3 mg, 17 µmol) and acrylic acid (1.1 µL, 16 µmol). Peptide-functionalised glass beads (60 g) were added to the monomer solution, which was then bubbled with nitrogen for 20 min. Polymerisation was initiated through the addition of ammonium persulphate (30 mg, 0.13 mmol) and *N,N,N’,N’*-tetramethyl ethylenediamine (30 µL, 0.2 mmol) in water (500 µL). The mixture was shaken and incubated for 1 h before being transferred to a solid phase extraction cartridge fitted with a 20 µm polyethylene frit. Unreacted monomers and low affinity polymers were removed from the glass beads by washing with water (5 × 100 mL). High affinity polymers were collected with hot ethanol (65 °C, 2 × 25 mL), reduced to 5 mL under vacuum and dialysed in water for 1 week using 12 kDa cellulose membranes with regular change of water.

### 2.7. Surface Plasmon Resonance (SPR) Measurement

Binding analysis was performed using a Biacore 3000 instrument (Cytiva, UK) at 25 °C using PBS (0.01 M phosphate buffer, 0.0027 M potassium chloride and 0.137 M sodium chloride, pH 7.4) as the running buffer at a flow of 35 µL min^−1^. The self-assembled gold sensor chip was plasma-cleaned using a K1050X RF Plasma Etcher/Asher/Cleaner barrel reactor (Quorum Technologies Ltd., Lewes, UK) and placed in a solution of mercaptododecanoic acid in ethanol (1.1 mg mL^−1^) where they were stored until use. Before assembly, the sensor chip was rinsed with ethanol and water and dried in a stream of air. Each cysteine-containing specific or scrambled peptide was immobilised *in situ* on the chip surface containing carboxyl groups using thiol coupling. First, the surface was activated using an EDC and NHS mixture (0.4 mg and 0.6 mg mL^−1^, respectively). 2-(2-pyridinyldithio)ethaneamine hydrochloride (PDEA, 80 mM) in 50 mM sodium borate buffer pH 8.5 was injected in order to introduce disulphide bonds; 100 µL of 10 µg mL^−1^ peptide solution in PBS was then injected at a 15 µL mL^−1^ flow rate followed by surface deactivation using cysteine/NaCl solution.

The peptide-specific nanoMIPs were briefly sonicated and diluted with PBS in the concentration range 0.04–1 nM. Sensorgrams were collected sequentially for all analyte concentrations running in KINJECT mode (injection volume—100 μL and dissociation time—120 sec). Dissociation constants (K_d_) were calculated from plots of the equilibrium biosensor response using the BiaEvaluation v 4.1 software using a 1:1 Langmuir binding model fitting after subtraction of drift and bulk components.

## 3. Results and Discussion

**Scheme 2:**
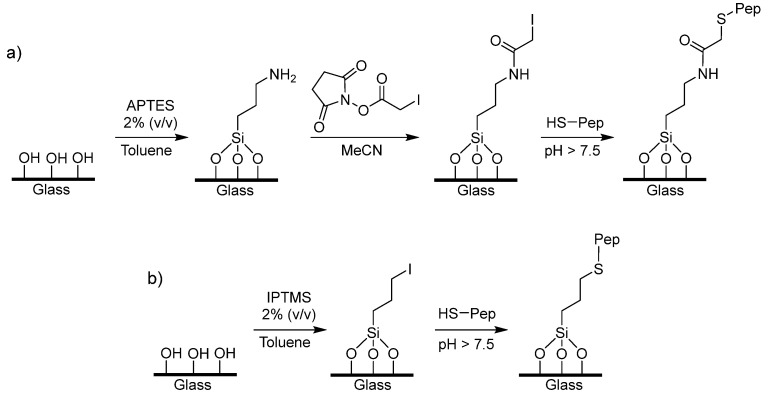
Immobilisation of peptides on glass beads using; (**a**) APTES; (**b**) IPTMS. ‘Pep’ refers to the EGFR peptide being immobilised.

This work investigates the replacement of amino silanes for the immobilisation of thiol-presenting templates—a common strategy for the imprinting of peptides and proteins. The immobilisation of peptides is commonly performed via conjugation of a terminal cysteine unit to an amino silane using a linker, such as SIA [7,13,17]. Herein we attempted to replace the amino silane and SIA linker with an iodo silane, (3-iodopropyl)trimethoxy silane (IPTMS) (Scheme 2). This serves three benefits: removal of unwanted side reactions caused by amine groups, reduction in the number of steps necessary for template immobilisation, and the replacement of relatively expensive SIA.

In order to compare the performance of these two approaches, peptide-functionalised glass beads were prepared using both AHAMTES and IPTMS-based protocols (Table 1). These solid phases were then used for the synthesis of MIPs via a solid phase approach as described by Canfarotta et al. [13]. The peptide selected for imprinting was an epitope of epidermal growth factor receptor (EGFR), a cancer biomarker of clinical interest. This sequence (KLFGTSGQK) was previously identified using a MIP-based epitope mapping technique [8]. The peptide was prepared with a terminal cysteine for immobilisation and an additional glycine to act as a spacer (full sequence CGKLFGTSGQK). A scrambled version of the peptide was also imprinted to act as a control (full sequence CGTKGKQLSGF). The density of peptides on the surface of the glass beads following immobilisation was determined via bicinchoninic acid assay (BCA). The density of immobilised peptide was similar in both cases, at 6.3 nmol peptide/g glass beads using AHAMTES and 6.1 nmol peptide/g glass beads using IPTMS. These values are similar to those previously reported for the immobilisation and imprinting of proteins including trypsin (1.7 nmol/g), pepsin A (2.8 nmol/g) and amylase (2.9 nmol/g) [18]. The difference of a factor of two can be explained by the larger size of these protein templates as compared to the peptides within this study.

**Figure 1:**
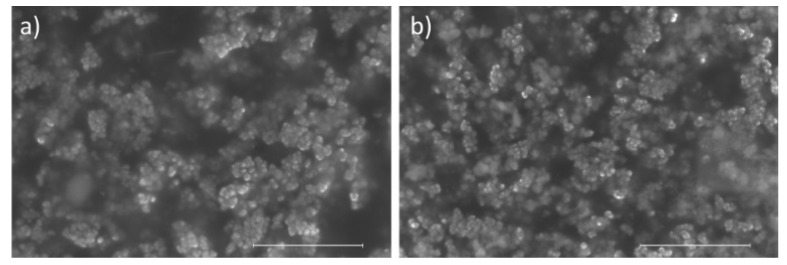
Scanning electron microscopy (SEM) images of MIPs prepared using: (**a**) AHAMTES-based solid phase, (**b**) IPTMS-based solid phase. Scale bar = 2 µm.

The amount of MIP nanoparticles collected following polymerisation, elution and dialysis was found to be 56 µg MIP/g glass beads using AHAMTES, and 72 µg MIP/g glass beads using IPTMS. The average size of these particles was found to be approximately 60 nm in both cases (Figure 1). The protocol for AHAMTES-functionalisation of glass includes a lengthy (overnight) washing step in order to remove weakly associated silane multilayers. This step is omitted in the IPTMS-based protocol. As shown in the results of elemental analysis, even in the absence of such a washing step the level of silane contamination is lower in the case of IPTMS-MIPs as compared to AHAMTES-MIPs (Tables S2 and S3). 

**Figure 2:**
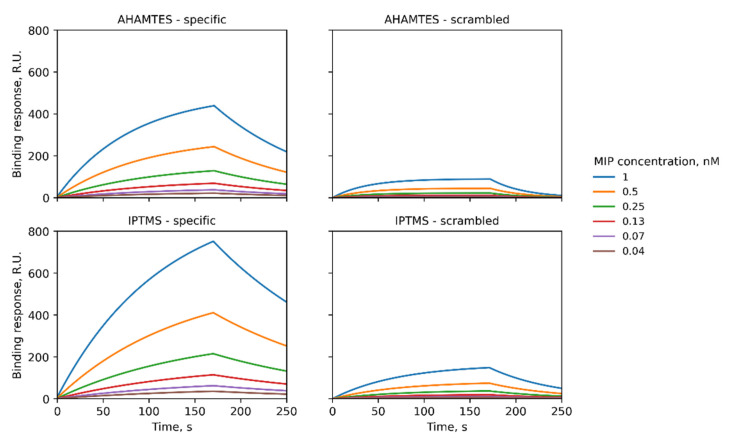
SPR binding curves of MIPs prepared using AHAMTES and IPTMS against specific and scrambled peptides.

The binding performance of MIPs prepared using both silanes was compared via surface plasmon resonance (SPR) measurement (Figure 2, Table 2). The specificity of the resultant MIPs was assessed by comparison of binding affinity with a scrambled version of the same peptide (CGTKGKQLSGF). In both cases, the peptide was immobilised on gold SPR chips, and MIPs prepared using both techniques were injected during SPR measurement.

Both MIPs prepared using AHAMTES and IPTMS showed excellent binding affinity (K_d_ of approximately 2 nM) for their specific peptide and significantly lower affinity (K_d_ of approximately 700 nM) for the scrambled control peptide (Table 2). The dissociation constant for the scrambled control peptide was marginally lower for MIPs prepared using AHAMTES as a solid phase as compared to those made using IPTMS. This may possibly be attributed to the slightly higher level of silane contamination increasing the level of non-specific binding between the MIPs and the gold SPR chip (Tables S2 and S3).

An additional advantage of IPTMS over amino silanes is the lack of residual amines on the surface of the glass beads following MIP formation. Post-synthetic labelling of MIPs is often performed using amine-reactive probes, such as NHS-ester-DyLight and AlexaFluor. In order to preserve binding site efficacy, this tagging can be performed while the MIPs are still bound to the solid phase, preventing the conjugation of probes to functional groups within the binding sites [19]. In this case, the use of amino silanes for template immobilisation would result in probes linking to any remaining amine groups of the solid phase, resulting in wasted (often expensive) probes. The use of iodo silanes circumvents this issue.

Finally, alkyl halides, such as IPTMS are able to react with other nucleophilic groups beyond thiols (notably amines and aromatic alcohols, given basic enough conditions). As a result, the iodo silane-based methodology described herein can be used for the immobilisation of a wide variety of template molecules even in the absence of cysteine groups. However, in the case of templates containing multiple nucleophiles the pH must be carefully controlled or protecting groups employed to ensure immobilisation via the intended functional group to reduce the variability of MIP binding sites.

## 4. Conclusions

Amino silanes commonly used for solid phase preparation were investigated and found to show undesired side reactions during solid phase synthesis of molecularly imprinted polymers (MIPs), including the formation of silane multilayers and reaction with persulphates. An iodo silane (IPTMS) is presented as an alternative for peptide immobilisation, and was compared to an amino silane (AHAMTES) for the solid phase synthesis of MIP nanoparticles specific for an epitope of epidermal growth factor receptor (EGFR). The iodo silane tested was found to equal or outperform the amino silane by each metric tested. Both protocols produced similar yields of MIPs with excellent affinity (K_d_ ≈ 2.5 nM), but with fewer experimental steps and lower cost reagents necessary for the iodo silane-based protocol.

## Figures and Tables

**Table 1:** Comparison of methodologies for AHAMTES and IPTMS-based solid phase preparation.

	AHAMTES	IPTMS
**Silanisation**	Incubate glass beads (60 g) in 2% (*v*/*v*) AHAMTES overnight in dry toluene (60 mL). Wash with acetone (4 × 100 mL). Dry at 120 °C for 30 min.	Incubate glass beads (60 g) in 2% (*v*/*v*) IPTMS overnight in dry toluene (60 mL), protected from light. Wash with acetone (4 × 100 mL). Dry at 120 °C for 30 min.
**Removal of multilayers**	Soak glass beads overnight in a large excess of water. Wash with acetone (4 × 100 mL).	-
**Surface activation**	Incubate silanised glass beads (60 g) in a solution of succinimidyl iodoacetate (SIA) (5 mg) in anhydrous acetonitrile (25 ml) for 2 h, protected from light. Wash with acetonitrile (5 × 50 mL).	-
**Peptide conjugation**	Prepare ethylenediaminetetraacetic acid (EDTA) (74 mg, 500 µmol, 5mM) in phosphate buffered saline (PBS, 10 mM, 50 mL), adjust to pH 8.2 with sodium hydroxide. Add SIA-functionalised glass beads (60 g) and EGFR peptide (5 mg), incubate overnight protected from light.	Prepare EGFR peptide (5 mg) in borate buffer (pH 9.2, 30 mM sodium tetraborate, 25 mL). Add IPTMS-functionalised glass beads (60 g) and incubate overnight, protected from light.
**Surface quenching**	Add mercaptoethanol (20 µL) to a mixture of glass beads and peptide, and incubate for 2 h protected from light. Wash with water (2 × 500 mL) and acetone (100 mL), and allow to dry.	Add mercaptoethanol (20 µL) to a mixture of glass beads and peptide, and incubate for 2 h protected from light. Wash with water (2 × 500 mL) and acetone (100 mL), and allow to dry.

**Table 2:** Dissociation constants (K_d_) of MIPs prepared using AHAMTES and IPTMS-functionalised glass against specific and scrambled peptide.

Silane	Peptide	K_d_ [nM] (χ^2^)
AHAMTES	Specific	2.5 (7.14 × 10^–5^)
AHAMTES	Scrambled	676 (1.58 × 10^–5^)
IPTMS	Specific	2.3 (7.14 × 10^–5^)
IPTMS	Scrambled	766 (2.06 × 10^–5^)

## Data Availability

Additional data available upon request from authors.

## Supplementary Materials

The following supporting information can be downloaded at: https://www.mdpi.com/article/10.3390/polym14081595/s1, S1. Additional Methods; S2. Additional Discussion; Figure S1. Silane density of glass beads coated with APTES and AHAMTES after various levels of washing [20]; Table S1: Contents of vials displayed in Figure S2 and results of gelation experiment [21]; Figure S2: Vials of acrylamide solution in which polymerisation was initiated with APS and various co-initiators; Figure S3: EDS mapping spectra of MIPs prepared using AHAMTES solid phase; Table S2: Elemental analysis of AHAMTES MIPs using EDS; Figure S4: EDS mapping spectra of MIPs prepared using IPTMS solid phase; Table S3: Elemental analysis of IPTMS MIPs using EDS.
