# Supplementary material for: Iodo Silanes as Superior Substrates for the Solid Phase Synthesis of Molecularly Imprinted Polymer Nanoparticles
Source: Polymers (Basel). 2022 Apr 14;14(8):1595. doi: 10.3390/polym14081595 (PMC9026888; doi:10.3390/polym14081595)
Supplement: Supplementary file 1 [file polymers-14-01595-s001.zip › polymers-1671374-supplementary.pdf]

Iodo Silanes as Superior Substrates for the Solid Phase Synthesis of Molecularly Imprinted Polymer Nanoparticles

Stanislav S. Piletsky,* Alvaro Garcia Cruz, Elena Piletska, Sergey A. Piletsky, Eric O. Aboagye and Alan C. Spivey

Supplementary Information

S1. Additional Methods

S1.1. Measurement of Amino Silane Density

Glass beads functionalized with APTES and AHAMTES were each divided into 4 batches of 7.5 g and soaked in 50 mL of water for different amounts of time (0 h, 5 min, 24 h and 72 h). They were then washed with acetone (2 x 50 mL) and dried at 120 °C for 30 min. A solution of ninhydrin (40 mg, 0.2 mmol) in ethanol (20 mL) was prepared. Solutions of AHAMTES in ethanol (2x dilution series, 25 mM to 0.2 mM) were prepared. The AHAMTES solutions (0.5 mL) were mixed with the ninhydrin solution (0.5 mL) and shaken at 60 °C for 90 min. 330 mg of each batch of glass beads were added to ethanol (0.5 mL) and ninhydrin solution (0.5 mL). These were also shaken at 60 °C for 90 min. 200 µL of each solution was then decanted and the absorbance measured at 570 nm using a Tecan Infinite M200 (Tecan, Reading, UK) in triplicate. The AHAMTES solutions were used to form a calibration curve in the linear region of 0.2 to 3.1 mM, and the density of silane on the glass beads was then calculated.

S1.2. Demonstration of APS-Amine Induced Initiation

A solution of 20% (w/w) acrylamide and 1% (w/w) *N,N′*-methylene bis(acrylamide) in water (10 mL) was prepared. Acryloxyethyl thiocarbamoyl rhodamine B (0.05% w/w) was added in order to help visualize gelation. 0.5 mL of this solution was decanted into 13 vials. These samples were treated with different amines and numbered as described in Table S1, then incubated at room temperature for 5 min.

S2. Additional Discussion

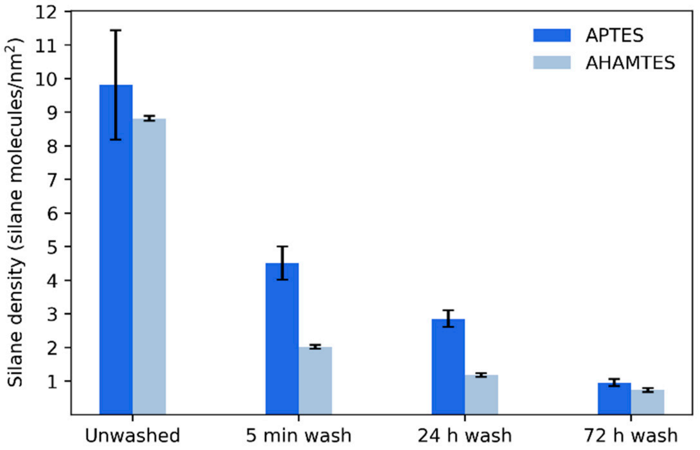

Figure S1. Silane density of glass beads coated with APTES and AHAMTES after various levels of washing.

In order to demonstrate the presence of amino silane multilayers, glass beads were silanized using APTES and AHAMTES, washed with water for varying lengths of time, and their density measured *via* reaction with ninhydrin. The density of an APTES monolayer on the surface of silica has previously been reported to be around 1.23 APTES molecules/nm^2, following a washing step to remove weakly bound silane [1]. As shown in Figure S1, the initial density of APTES and AHAMTES on the surface of glass prior to any washing was significantly higher than this (9.8 and 8.8 silane molecules/nm^2, respectively). After prolonged soaking, the silane density of APTES and AHAMTES decreases to a minimum of 1.0 and 0.7 silane molecules/nm^2, respectively. This indicates the presence of weakly bound silane molecules which are hydrolyzable under the conditions used for both template immobilization and MIP synthesis.

Acrylamide radical polymerization is commonly initiated using a combination of ammonium or potassium persulfate (APS/KPS) and *N*′-tetramethyl ethylenediamine (TEMED). TEMED and other tertiary amines accelerate the rate of APS/KPS decomposition, allowing for room temperature polymerization. Polymerization was initiated *via* addition of APS alone, APS and TEMED, or APS and two commonly used amino silanes (APTES and AHAMTES). A small amount of rhodamine-acrylate (0.05 % w/w) was added to help visualize gelation. Polymerization was carried out for 5 min at room temperature (Figure S2, Table S1).

In the presence of TEMED and APS, the solution rapidly polymerized, forming a thick gel (Figure S2, vial 2). Similarly, the solutions formed a gel in the presence of APS and either amine-presenting silane (Figure S2, vials 4 and 6), along with propylamine (to demonstrate that initiation is induced by the amine functionality) (Figure S2, vial 8). No visible gelation occurred over this timeframe following the addition of APS or either of the silanes or propylamine alone (Figure S2, vials 1, 3, 5 and 7). The presence of (2,2,6,6-tetramethylpiperidin-1-yl)oxyl (TEMPO) as a radical trap inhibited gelation, confirming that polymerization is proceeding *via* a radical mechanism (Figure S2, Table S1).

TEMED serves two roles within acrylamide polymerization: increasing the rate of persulfate decomposition, and acting as an anchor from which the polyacrylamide chain grows [2]. Solid phase MIP synthesis involving amino silanes capable of acting as anchors runs the risk of the resulting polymers becoming covalently linked with the solid phase itself, resulting in difficulty in eluting the resultant polymers from the solid phase. In this work, we did not explore the precise mechanism by which amino silanes trigger polymerization, and so we cannot comment on the extent to which this

anchoring occurs. It is possible that this anchoring can prove beneficial for solid phase MIP synthesis. By using amino silanes as a co-initiator of APS in place of TEMED, the majority of polymerization will occur at the interface between the solid and liquid phases, in close proximity to the template. However, this has yet to be explored in any detail, and currently exists only as an uncontrolled side-reaction along with traditional TEMED-based initiation.

Table S1. Contents of vials displayed in Fig S2 and results of gelation experiment.

Number	Amine	APS	TEMPO	Gelation
1	-	5 mg	-	×
2	TEMED (5 μL)	5 mg	-	✓
3	APTES (30 μL)	-	-	×
4	APTES (30 μL)	5 mg	-	✓
5	AHAMTES (40 μL)	-	-	×
6	AHAMTES (40 μL)	5 mg	-	✓
7	Propylamine (11 μL)	-	-	×
8	Propylamine (11 μL)	5 mg	-	✓
9	-	-	0.5 mg	×
10	TEMED (5 μL)	5 mg	0.5 mg	After 15 min incubation
11	APTES (30 μL)	5 mg	0.5 mg	After overnight incubation
12	AHAMTES (40 μL)	5 mg	0.5 mg	After overnight incubation
13	Propylamine (11 μL)	5 mg	0.5 mg	After overnight incubation

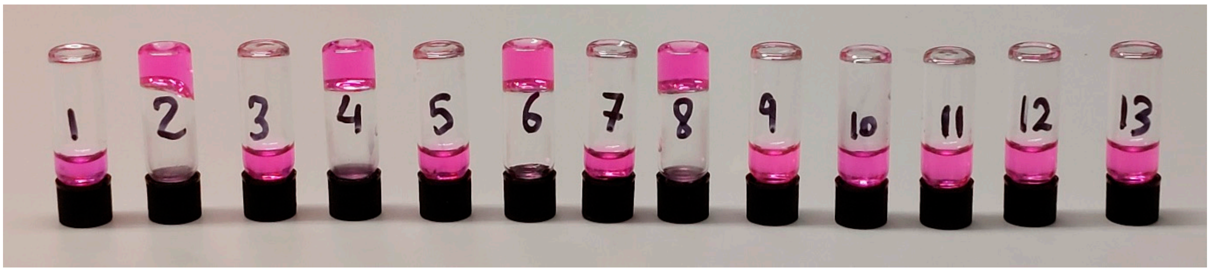

Figure S2. Vials of acrylamide solution in which polymerization was initiated with APS and various co-initiators, 5 min after initiation.

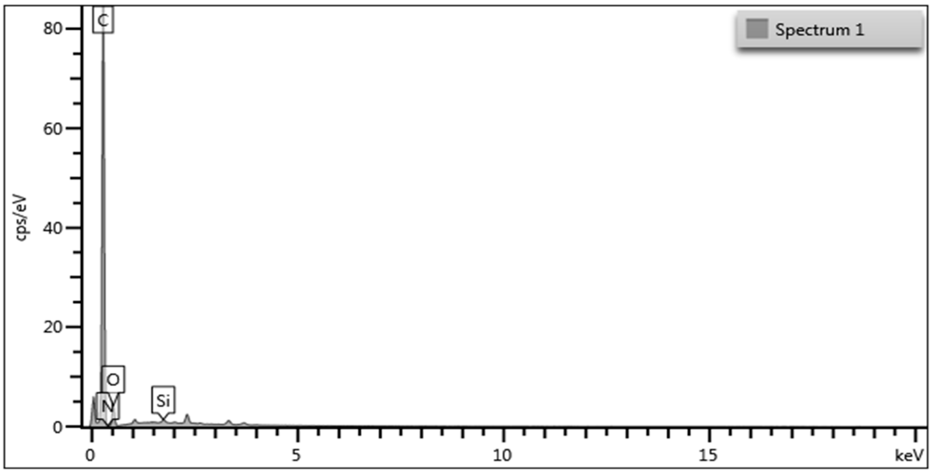

Figure S3. EDS mapping spectra of MIPs prepared using AHAMTES solid phase.

Table S2. Elemental analysis of AHAMTES MIPs using EDS.

Element	Line Type	Wt%	Wt% Sigma
C	K series	83.93	0.19
N	K series	0.00	1.00
O	K series	15.81	0.19
Si	K series	0.26	0.02

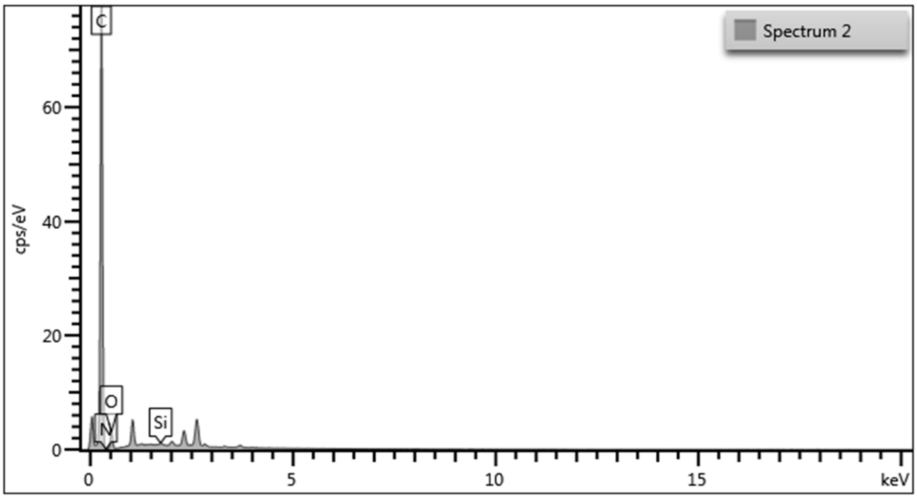

Figure. S4. EDS mapping spectra of MIPs prepared using IPTMS solid phase.

Table S3. Elemental analysis of IPTMS MIPs using EDS.

Element	Line Type	Wt%	Wt% Sigma
C	K series	86.21	0.20
N	K series	0.00	1.10
O	K series	13.65	0.20
Si	K series	0.14	0.02

Additional References

1. Cuoq, F.; Masion, A.; Labille, J.; Rose, J.; Ziarelli, F.; Prelot, B.; Bottero, J.Y. Preparation of Amino-Functionalized Silica in Aqueous Conditions. Applied Surface Science 2013, 266, 155–160, doi:10.1016/J.APSUSC.2012.11.120.
2. Gómez-Caballero, A.; Elejaga-Jimeno, A.; García del Caño, G.; Unceta, N.; Guerreiro, A.; Saumell-Esnaola, M.; Sallés, J.; Goicolea, M.A.; Barrio, R.J. Solid-Phase Synthesis of Imprinted Nanoparticles as Artificial Antibodies against the C-Terminus of the Cannabinoid CB1 Receptor: Exploring a Viable Alternative for Bioanalysis. Mikrochimica Acta 2021, 188, 3, doi:10.1007/S00604-021-05029-Z.
